# Short-term effect of osteopathic manual techniques (OMT) on respiratory function in healthy individuals

**DOI:** 10.1371/journal.pone.0235308

**Published:** 2020-06-30

**Authors:** Jakub Stępnik, Agnieszka Kędra, Dariusz Czaprowski

**Affiliations:** 1 Still Academy of Osteopathy, Warsaw, Poland; 2 Faculty of Physical Education and Health, Józef Pilsudski University of Physical Education in Warsaw, Biała Podlaska, Poland; 3 Department of Health Sciences, Physiotherapy Unit, University of Medical Science, Poznań, Poland; 4 Department of Health Sciences, Olsztyn University, Olsztyn, Poland; University of Pisa, ITALY

## Abstract

**Background:**

Respiratory system diseases are some of the most common pathologies worldwide. Although osteopathic manual therapy (OMT) is used predominantly to treat other pathologies, certain OMT techniques have been shown to improve patients’ respiratory function.

**Objectives:**

The aim of this study was to assess the influence of osteopathic techniques on breathing.

**Methods:**

Tests were performed with the use of a spirometer and the results were expressed as Forced Vital Capacity (FVC), Forced Expiratory Volume in 1 second (FEV1), and Peak Expiratory Flow (PEF). Thirty healthy males and females between the age of 18 and 50 took part in the research. Fifteen individuals were randomly assigned to the experimental group and fifteen persons were assigned to the placebo group. The participants from the experimental group were treated with such osteopathic techniques aimed at the pulmonary system as the thoracic thrust (manipulations of vertebral joints and ribs), the sternal pump technique and stretching of the diaphragm. The placebo group was treated with soft tissue therapy (STT) techniques for the masseter muscle.

**Results:**

The described set of osteopathic techniques exerts an influence on PEF in healthy individuals; however, it does not affect FVC and FEV1.

**Conclusion:**

Osteopathic techniques do not seem to improve lung health, as reflected in FEV1 and FVC, but they improve the respiratory function aspects reflected by PEF in the participants without any history of lung disease.

## Introduction

Breathing is one of the most vital biological functions of the body that ensures the continuum of life. Without oxygen, human beings cannot live for more than a few minutes. Pathologies of the respiratory system are some of the biggest problems modern medicine faces today. According to WHO statistics, these pathologies occupy the sixth place among the most common causes of death in the world and it is expected that by 2030 it will have taken the third place [1]. Typical symptoms of pulmonary diseases are dyspnoea, coughing, depression/anxiety, exhaustion and pain [2].

The osteopathic manual therapy (OMT) approach is not traditionally seen as part of standard treatment of pulmonary diseases but each year more and more researchers conduct studies in this area. Presently, OMT could serve as an adjunctive therapy for respiratory problems with a potential impact on the mobility of the thorax (the intervertebral joints, the costovertebral joints, bony structure, all ligaments, muscles, and fascia) and innervation of the lungs, pleura, diaphragm etc. [3].

Most often, studies in this area focus on the effects of OMT on patients suffering from COPD (Chronic Obstructive Pulmonary Disease) [1,4–6]. Nevertheless, specific soft tissue techniques used for treating COPD patients have not shown any statistically significant effects on lung function or on the level of dyspnoea. However, Barinow-Wojewódzki [6] wrote about a positive influence of the rehabilitation program on lean body mass and water level in COPD patients. Asthma is another similar area of research in which the same treatment approach is used [7–10]. Guiney et al. [7] noted that pediatric patients with asthma who underwent OMT treatment experienced statistically significant improvement in pulmonary function measured in PEF compared to the control group. Lowhagen and Bergqvist [11] observed that after soft tissue therapy and breathing exercises in asthma patients, PEF parameter improved significantly and such symptoms as ‘tightness in the chest’, ‘difficulty breathing in’ and ‘air hunger’ were significantly reduced in the active group.

The purpose of this study was to estimate the effects of using a combined set of both manipulative and soft OMT techniques on the respiratory system. The manipulative techniques included manipulations of the intervertebral and costovertebral joints, while the soft techniques included all fascial and muscle techniques. Several studies investigated the effects of either soft techniques [3,12] or manipulative techniques [8] on respiratory function. The present study combined both manipulative and soft techniques for potentially increased efficacy.

## Materials and methods

### Population

A sample group consisting of thirty volunteers aged 19–46 years was randomly divided into an experimental group and a placebo group. The experimental group included 15 females aged 19–42 years (mean 33.3 ± 5.7), while the placebo group consisted of 15 females aged 25–46 years (mean 33.4 ± 6). The duration of the study was 6 weeks.

#### Exclusion criteria

The exclusion criteria were as follows: males, high temperature of the body, lung problems (COPD, asthma, lung cancer, lung infection, pulmonary edema, pneumothorax bronchitis, influenza, tuberculosis, cystic fibrosis, sarcoidosis, pulmonary embolism), heart problems (arrhythmia, coronary artery disease, mitral regurgitation, congenital heart disease, hypertrophic heart disease, high blood pressure—over 140/90), acute visceral problems, known allergies, chronic respiratory diseases or acute illnesses in the last 3 months, respiratory or heart-related drugs taken in the last 3 months, previous surgery in the thorax area, vertigo, headache, pregnancy or any known contraindications to manipulation [13–15]. The participants who met these criteria were excluded from the study, while the remaining individuals were randomly assigned to the placebo or experimental group.

Each participant’s written consent was obtained prior to the commencement of the study. The Ethical Committee of Józef Rusiecki University College in Olsztyn granted permission for this research (permission number: 2/2016).

### Randomization

Thirty sealed envelopes were prepared, where 15 contained the letter ‘I’ representing the experimental group, and 15 contained the letter ‘P’ representing the placebo group. Allocation to the two groups was randomized and concealed from all the participants, as each participant randomly selected a sealed envelope from the set of prepared envelopes to be assigned to a particular group.

### Intervention applied in the experimental group

Intervention applied in the experimental group was performed with the use of 3 techniques:

#### Supine thoracic thrust manipulation [15]

The participants were asked to adopt a supine position. They were told to grab their shoulders with their opposite hands, with elbows pointed downwards. The therapist approached the participant from the left side and rolled her towards himself, putting his left hand under the spine, using what is called ‘a pistol grip’. To perform this, the participant’s head was turned to the left and she was rolled only to the point when the therapist could slip his hand under the spine. Then, using his hand, the therapist fixated the vertebra underneath. The therapist tested joint play in the direction of traction. Pressure was applied to this segment until the optimal joint capsule tension was reached. Thrust was performed by the therapist placing his right hand across the participant’s left arm and thorax and applying traction by pushing the participant’s elbows toward the shoulders.

#### Sternal pump and sternal recoil [16]

Sternal pump was also performed with the participants in the supine position. The therapist stood behind the participant’s head and placed both hands on top of each other on the participant’s sternum. The participant was asked to inhale deeply. During the deep exhalation, pressure was applied with both hands in the caudal and posterior direction. The pressure was released during the next inhalation. This procedure was repeated five times. The last two times were performed with a recoil.

#### Diaphragm stretch in a sitting position [3]

For the diaphragm stretch, the participant assumed a sitting position with the therapist standing behind. The therapist placed both hands underneath the lower part of the participant’s costal border grabbing their whole costal margin. Both hands followed the inspiratory movements of the ribs and stopped the ribs from returning during exhalation. While the participant inhaled, the therapist followed the movement. When the participant exhaled, the therapist resisted the thoracic border by pulling mildly until the feeling of resistance. This position was maintained, and the procedure was repeated several times at different points on the thoracic border.

#### Placebo intervention in the control group

An intervention procedure in the placebo group was performed with the use of STT (soft tissue therapy) for the masseter muscle [17]. The participants received the following explanation: “one of the osteopathic treatment approaches is to balance the diaphragms in the body”. There are 6 diaphragms in the human body, including the abdominal diaphragm and the temporomandibular joint. By balancing one diaphragm, it should be possible to affect other diaphragms, especially the diaphragm muscle which has a direct influence on the lungs and is the most important respiratory muscle. Basic spirometry was performed to evaluate the value of the procedure.

*Description of the technique*. The procedure was performed with the participants in a prone position. Head support was modified according to the participants’ needs, as close as possible to a neutral position. The therapist was in a sitting position behind the participant’s head, performing friction techniques on the masseter muscle and longitudinal stretch until tenderness and local spasm decreased. There is no direct influence of this muscle on the lungs; therefore, this muscle was chosen.

All the techniques were performed by an osteopath who is simultaneously a physiotherapist with 10 years of clinical experience. Additionally, all the techniques were consulted with two osteopaths with at least 20 years of experience in osteopathic treatment.

### Spirometry device

The spirometry test was performed using a pneumotachometer, a spirometry device from Contec Medical System CO, China, type SP 10.

#### Spirometry protocol [2]

Prior to the test, the participants were instructed how to perform a correct spirometry test using maximal exhalation effort. All the participants received clear and continuous support during all tests. The testing was performed with the participants wearing only underwear, in a sitting position with the head held in a neutral position. The tests were always performed in the same place at room temperature. The spirometry test was performed both before and after the treatment procedure in both groups.

#### Preparation of the participants [2]

The tests began with the participants performing trial tests to improve their breathing pattern. They were asked to repeat the tests until 3 well-performed tests were executed to ensure that the repeatability criteria were met. Each participant’s single best-performed measurements were kept for analysis [2].

#### Spirometry parameters

In this study, inspiratory as well as expiratory variables were expressed in absolute values and noted as the percentage of reference value. The inspiratory and expiratory variables were as follows: FVC (forced vital capacity) expressed in L, FEV1 (forced expiratory volume in 1 second) expressed in L and PEF (peak expiratory flow) expressed in L/sec.

### Procedure

The variables tested by spirometry included FVC expressed in litres, FEV1 expressed in litres and PEF expressed in litres per second. The osteopathic intervention consisted of a set of manual techniques aimed at influencing lung function. The placebo intervention involved STT that targeted the masseter muscle. Neither participants nor statisticians were aware of who was selected to which group.

### Statistical analysis

The parameters were described using basic descriptive statistics measurements, i.e., median, minimum, maximum, 1^st^ and 3^rd^ quartile. Descriptive statistics were calculated separately for both experimental and placebo groups. The compliance of the results with the normal distribution was checked with the use of the Shapiro-Wilk test. Wilcoxon signed rank test was used to investigate differences in spirometry parameters before and after OMT. To examine differences between the experimental group and the placebo group, the Mann-Whitney U test was applied to compare values before and after OMT. Statistical significance was set at p<0.05. The collected material was organized and analyzed using the Statistica 13.3 calculation software by Statsoft (Poland).

## Results

There were no significant differences between experimental and placebo groups in pulmonary parameters assessed prior to OMT and STT (Table 1).

**Table 1:** The pulmonary parameters in experimental and placebo groups before the study.

Parameter	Group	Median	Min	1st Qu	3rd Qu	Max	*p*
FVC	Experimental	4.0	2.5	2.8	4.9	5.3	0.66
	Placebo	3.9	2.5	3.6	4.2	5.7	
FEV1	Experimental	3.7	2.1	2.7	4.1	4.6	0.48
	Placebo	3.5	1.8	3.2	3.6	4.5	
PEF	Experimental	7.1	2.8	5.5	8.4	11.2	0.18
	Placebo	8.2	5.1	7.0	9.3	11.9	

FVC—Forced Vital Capacity; FEV1—Forced Expiratory Volume in 1 second; PEF—Peak Expiratory Flow

There were no significant differences regarding FVC and FEV1 observed before and after OMT in the experimental group and no significant differences concerning STT in the placebo group. PEF significantly increased only after OMT in the experimental group (p<0.001) (Table 2).

**Table 2:** Pulmonary parameters before and after OMT and STT observed in experimental and placebo groups.

Parameter	Group		Median	Min	1st Qu	3rd Qu	Max	*p*
FVC	Experimental	Before	4.0	2.5	2.8	4.9	5.3	0.07
		After	4.5	2.7	3.2	5.1	5.7	
	Placebo	Before	3.9	2.5	3.6	4.2	5.7	0.98
		After	4.2	2.6	3.6	4.4	5.5	
FEV1	Experimental	Before	3.7	2.1	2.7	4.1	4.6	0.14
		After	3.9	2.5	2.8	4.3	4.5	
	Placebo	Before	3.5	1.8	3.2	3.6	4.5	0.3
		After	3.5	1.9	3.4	3.7	4.6	
PEF	Experimental	Before	7.1	2.8	5.5	8.4	11.2	**<0.001**
		After	8.1	4.8	6.7	9.3	12.2	
	Placebo	Before	8.2	5.1	7.0	9.3	11.9	0.95
		After	8.7	5.2	7.0	9.4	11.9	

OMT—Osteopathic Manual Therapy; STT—soft tissue therapy; FVC—Forced Vital Capacity; FEV1—Forced Expiratory Volume in 1 second; PEF—Peak Expiratory Flow; significant difference was marked in bold

There were no significant differences between the experimental group and the placebo group with regard to pulmonary parameters evaluated after OMT and STT (Table 3).

**Table 3:** Pulmonary parameters in experimental and placebo groups after OMT and STT.

Parameter	Group	Median	Min	1st Qu	3rd Qu	Max	*p*
FVC	Experimental	4.5	2.7	3.2	5.1	5.7	0.32
	Placebo	4.2	2.6	3.6	4.4	5.5	
FEV1	Experimental	3.9	2.5	2.8	4.3	4.5	0.43
	Placebo	3.5	1.9	3.4	3.7	4.6	
PEF	Experimental	8.1	4.8	6.7	9.3	12.2	0.87
	Placebo	8.7	5.2	7.0	9.4	11.9	

OMT—Osteopathic Manual Therapy; STT—soft tissue therapy; FVC—Forced Vital Capacity; FEV1—Forced Expiratory Volume in 1 second; PEF—Peak Expiratory Flow

## Discussion

The effects of osteopathic and manual therapy treatment on respiratory function is a very broad subject of research. There are studies that have shown positive effects on patients [3,4,6,8,11,12,18], but there are no established standards or clear guidelines for the use of osteopathic and manual therapy (OMT).

For our treatment protocol, four osteopathic manual techniques were used which aimed to improve respiratory function: sternal pump-recoil, manipulation of the thoracic intervertebral joints and the costotransversal joints, diaphragm mobilization and rib mobilization. The thoracic thrust techniques and ribs mobilization performed on the upper thoracic spine T1-T5 may have a normalizing effect on the sympathetic innervation of the lungs by the pulmonary plexus sympathetic chain, located closely to the rib heads. These techniques may affect joint mobility of the thoracic cage, which is very important for proper respiratory function. Without sufficient mobility of each joint of the chest, expansion of the lungs is likely to be reduced or limited.

The recoil technique, which is performed on the sternum, may increase the mobility of the cartilaginous connections between the sternum and the ribs as well as improve lung expansion.

Stretching of the diaphragm was considered the most important technique in this set, as the diaphragm is the most important respiratory muscle with direct influence on lung function. Proper function of the diaphragm (contraction and release) has a major influence on inspiratory and expiratory capacity and the mobility of the rib cage, especially in its lower parts. Each technique used in the chosen set is known to have either a direct or indirect influence on respiratory function. The majority of authors use manipulation techniques and diaphragm mobilization, two most popular osteopathic techniques employed to improve respiratory function [4,8].

Research has shown that a combination of different osteopathic techniques which include joint manipulations, joint mobilizations as well as soft tissue and cranial techniques is more effective than using only one technique [4,9,18]. We would suggest further investigations follow this multi-technique approach for increased effectiveness and results. PEF parameter levels seem to be the best and most obvious methods to evaluate the effects of such osteopathic treatments. Spirometry is the most typical tool for measuring respiratory function and is used by most authors [3,4,7,8,19]. Another common tool is a questionnaire [9].

Mehl-Madrona et al. [9] conducted a comparative randomised control trial on 89 individuals with asthma (68 completed the study) and compared four groups, i.e., the first group that underwent 12 sessions of cranial osteopathy, the second group that had 12 sessions of acupuncture, the third group that received a combination of both, i.e., 6 sessions of cranial therapy and 6 sessions of acupuncture, and the control group. The evaluated variables included the lung function test, the Asthma Quality of Life questionnaire, medication use as well as depression and anxiety levels. The results showed no statistically significant differences in pulmonary function tests for all groups. The quality of life increased and drug usage was reduced in the treatment group, but the levels of depression and anxiety remained unchanged.

Despite these weak study results, there is some evidence supporting the hypothesis that specific techniques of osteopathic treatment can be beneficial for patients with lung problems. One direct benefit is relaxation of any spinal dysfunction causing irritation of autonomic and somatic nerves contributing to muscle stiffness and pain [18]. Some authors claim that mechanical correction of vertebral subluxation by joint manipulation can restore normal function of the joint and nerve function and should therefore improve airway function as well [10]. Cranial osteopathy has also been found to be beneficial for the parasympathetic flow in patients with disturbances of the respiratory system [9].

Abdelaal Ashraf et al., studied 195 male patients with COPD [4]. They created a 12-week protocol, including two sessions per week to study ventilatory functions (VF) and functional capacity (FC) responses to diaphragmatic manipulation, costal manipulation or both. Each group showed statistically significant improvement, although the group with the combined diaphragmatic and costal manipulation treatments had the best results.

A statistically significant improvement in PEF was observed in the participants who received osteopathic manipulative therapy [19]. This positive effect was attributed to several factors: a decrease in the levels of anxiety, changes in the overall autonomic nervous system that have caused airway relaxation and balanced muscle tone and, what is particularly significant, an improvement in mechanical chest mobility.

Swender et al. [3], investigated the effects of osteopathic manual therapy in a group of 33 patients diagnosed with cystic fibrosis. Their manipulative protocol included rib rising, abdominal diaphragm release, thoracic inlet myofascial release, thoracic lymphatic pump and suboccipital decompression. No changes were found between the OMT group and the control group with spirometry of FEV1%. There were significant changes between both groups in the questionnaire on breathing, pain and anxiety. Fifteen out of 16 patients from the OMT group showed an improvement, whereas only 8 out of 16 individuals in the control group reported a positive change.

A combination of soft tissue techniques and joint manipulations had a short-term, though statistically significant effect on dyspnoea level and exercise performance [5]. This suggested the importance of the innervating value of the manual approach for patients with lung problems.

Most researchers have performed short-term studies without tracking long-term results and only a few studies have been conducted on large groups of participants [18]. There is no clear consensus in the literature regarding non-pharmacological methods of treatment for asthma symptoms; however, osteopathy offers a valuable alternative approach, as research has shown the benefits of osteopathic manipulative treatment in clinical conditions treating diseases such as asthma.

### Limitations

There are some limitations in this study. Firstly, it involved only one session per one participant. Naturally, extended treatment and study should give more significant results. It is estimated that an exchange of collagen fibers in the fascia and an improvement in other parameters take 9 months. Secondly, other techniques such as visceral manipulation, which is directly connected with the intercostal muscle fascia, could have been included in the study to focus more directly on the pleura of the lungs [20]. Another limitation was a small number of study participants. An estimated test group for the assumed test power should be 164 persons for relevant tests, which will be considered by the authors in the next study.

It is also worth noting that there is a lack of strong evidence for osteopathic treatment in lung conditions. Only few studies revealed certain benefits of osteopathic manipulative techniques, which could be part of the therapy of the patients with pulmonary diseases together with pharmacology, surgery, physiotherapy, occupational therapy and a diet [3,4,5,9,18,19]. Therefore, at present it seems impossible to prepare specific standard protocols for osteopathic manipulative therapy in lung disorders, and further studies are needed in this area.

### Clinical relevance

This study showed that this specific set of osteopathic manual techniques has limited effects on respiratory function. To obtain significant positive effects, a combination of manipulative techniques such as manipulations of the intervertebral joints and the costovertebral joints combined with soft techniques including a diaphragm stretch, myofascial releasing of the cervical and thoracic region and sternal pump or visceral manipulations should be used.

## Conclusions

The respiratory-oriented osteopathic techniques have little or no influence on FVC or FEV1 levels in healthy individuals. However, the results of the study show a positive influence on PEF in healthy adults, indicating that PEF may be considered a reliable marker for measuring the effects of osteopathic manipulative treatments targeting the respiratory system. However, given the small number of the study participants, we suggest that further research be carried out to verify the relationship between osteopathic techniques and respiratory function.

## Supporting information

S1 Dataset (XLSX)

## Abbreviations

COPD: Chronic Obstructive Pulmonary Disease
FEV1: Forced Expiratory Volume in 1 second
FVC: Forced Vital Capacity
OMT: Osteopathic Manual Techniques
PEF: Peak Expiratory Flow
STT: Soft Tissue Therapy
WHO: World Health Organization
